# Neuroprotective and antimalarial effects of *Juglans regia* leaf extracts in a murine model of cerebral malaria

**DOI:** 10.3389/fvets.2025.1537686

**Published:** 2025-04-07

**Authors:** Afra Alharbi, Shurug Albasyouni, Esam Al-Shaebi, Saleh Al Quraishy, Rewaida Abdel-Gaber

**Affiliations:** Department of Zoology, College of Science, King Saud University, Riyadh, Saudi Arabia

**Keywords:** malaria, *Plasmodium berghei*, biological criteria, cerebral alterations, neurotransmitters

## Abstract

**Background:**

Malaria is a major public health problem caused by the apicomplexan *Plasmodium* parasite. Cerebral malaria (CM) is the most critical outcome of *Plasmodium* infection. It is becoming more difficult to manage, particularly in areas of multi-drug resistance. Scientists are focused on identifying alternative strategies to combat malaria infection. Therefore, this study was designed to evaluate the activity of *Juglans regia* leaf extract (JRLE) in *Plasmodium berghei*-infected C57BL/6 mice.

**Methods:**

The *J. regia* leaf extract (JRLE) was prepared using methanol and characterized by Fourier-transform infrared spectroscopy (FT-IR). Female C57BL/6 mice were divided into six groups (5 mice/group): control, non-infected but JRLE-treated (500 mg/kg), *P. berghei*-infected non-treated, and *P. berghei*-infected treated with JRLE (250 or 500 mg/kg) or chloroquine (10 mg/kg). Groups (3–6) were infected intraperitoneally with *P. berghei* (1 × 10⁵). Treatment (oral JRLE or chloroquine) was administered for 5 days starting on day 4. Parasitemia, survival, and body weight were assessed, and brains were collected on day 9 p.i. for histopathological analysis (H&E staining) and GFAP immunohistochemistry. GABA, glutamate, neurotransmitters (epinephrine, norepinephrine, dopamine, serotonin), and mRNA expression of signaling genes (Chrnb2, Gabbr1, Gnai1, Gria2) were evaluated using ELISA and real-time PCR.

**Results:**

Phytochemical screening by FT-IR demonstrated the presence of 10 functional groups in the JRLE. By day 9 after infection with the *P. berghei* parasite, the parasitemia was significantly reduced after JRLE treatment with a dose of 500 mg/kg (6.33% ± 1.18%) compared to the infected group (23.84% ± 2.06%) with a positive correlation with body weight. Our data showed that JRLE prolonged the survival curve of the infected mice. JRLE ameliorates the reduction of the brain index caused by *P. berghei* infection. Furthermore, histological analysis showed that infection with *P. berghei* exacerbates brain damage as evidenced by degeneration of Purkinje cells, cerebral hemorrhage, intravascular sequestrations of parasitized red blood corpuscles (pRBCs), and infiltration of lymphocytes. At the same time, treatment with JRLE mitigates the brain injury induced by the infection. JRLE reduced the level of GFAP expression in the brain tissue of the infected mice. Additionally, treatment with JRLE ameliorates the brain neurotransmitter disbalance (i.e., epinephrine, norepinephrine, dopamine, and serotonin) after *Plasmodium* infection. Upon JRLE treatment, Chrnb2, Gnai1, and Gabbr1 mRNA expression were down-regulated in the brain tissues derived from infected female C57BL/6 mice. Meanwhile, mRNA expression of Gria2 was up-regulated after JRLE inoculation. Our study proved that JRLE significantly ameliorated the neurotransmitter markers by increasing GABA levels and decreasing the glutamate level in the brain of *P. berghei*-infected mice.

**Conclusion:**

Taken together, the data reported here illustrate that *J. regia* leaf extracts possess potent antimalarial effects and may offer a potential drug lead for developing a safe, effective, and affordable antimalarial therapy. Further studies are recommended to include the broader organ-specific effects of plant extract.

## Introduction

Malaria is a life-threatening malady caused by mosquito-borne parasites belonging to the genus *Plasmodium* (phylum Apicomplexa) that have complex life cycles involving two hosts (female *Anopheles* mosquito and vertebrate host) (1). Four types of *Plasmodium* cause malaria in humans (*Plasmodium ovale*, *Plasmodium vivax*, *Plasmodium falciparum*, and *Plasmodium malariae*) (2). Among these four types, the *P. falciparum* that causes malaria *falciparum* is the most dangerous type because it can cause serious complications that lead to death (3). Rodent-specific *Plasmodium* species including *Plasmodium berghei*, *Plasmodium yoelii*, *Plasmodium chabaudi*, and *Plasmodium vinckei*, are commonly used in mouse models to study malaria (4).

Despite significant progress in the fight against malaria, about a billion people worldwide are at risk of malaria infection (5). In 2022, a total of 241 million cases of malaria and 627,000 deaths were reported globally (6). In Saudi Arabia, the highest malaria rates are mostly found in the southwestern parts of the country with sporadic malaria occurrence (7). The outcome of the *Plasmodium* infection can vary from a symptomatic infection to mild febrile disease, to severe malaria, the deadliest form of which is cerebral malaria (CM) (8). CM is the most severe neurological complication of malaria, and cognitive and behavioral deficits are commonly reported in surviving cases (9). One of the major hypotheses in CM is the sequestration of parasitized erythrocytes (pRBCs) in the cerebral microvascular endothelium leading to flow obstruction and decreased tissue perfusion, thereby compromising the function of the central nervous system (10). Due to the high degree of reproducibility, easily manageable characteristics, and development of histopathological and neurological signs typical of human CM, the murine model using *P. berghei* has been widely used to understand this condition better (11).

Active malaria chemotherapy helps to treat malaria in conjunction with blocking the vector’s parasite infectivity by exploiting the metabolic differences between *Plasmodium* and the host. Factors that include parasite resistance and unspecific drug toxicity restrict the therapeutic efficacy of the drugs (12). Antimalarial drugs may be divided into causal prophylaxis (act on the pre-erythrocytic forms), gametocytocides (act on gametocytes), sporontocides (inhibit the sporogonic phase of development of the parasite in the mosquito), and schizontocides (act on the asexual erythrocytic forms of all species of malaria parasites) (13). Chloroquine (CQ) is a schizontocidal drug that is strong and rapid for treating and preventing clinical symptoms (14).

Owing to the presence of antiparasitic drug resistance, experts are looking to find alternate sources of protection (15). Current frontline antimalarial drugs are either derived directly from plants or are synthetically produced from a plant-derived chemical compound as a template (16). Several studies have been conducted on various plants to find new natural antimalarial medicine in Saudi Arabia (17–20). The reliance on herbal medicine is mainly attributed to factors such as affordability, availability, and accessibility (21). Increasing research data has demonstrated the potential of herbal drugs owing to the many bioactive compounds they contain (22).

The studied plant, *Juglans regia*, belongs to the genus *Juglans* (family Juglandaceae). Walnuts (*J. regia*) have a significant economic value and medicinal importance for human health because of their biochemical composition of polyunsaturated fatty acids (23), fiber, minerals (potassium, calcium, and magnesium) (24), amino acids and proteins (25), and phenolic compounds and saponins (26–28). Among *J. regia* parts, leaves are highly effective in acting as an alternative therapy. Ethnopharmacological studies of walnuts indicate that it is a rich source of phytochemicals that shown to have therapeutic effects, including gastro-protective (29), antibacterial (30), anticancer (31), hepatoprotective and anti-inflammatory (32), antioxidant (33), anti-diabetic (33), antimicrobial (34), and antifungal (35). Based on the above ethnobotanical and bio-activity studies, the current study aims to investigate the *in vivo* anti-malarial effect of the methanolic *J. regia* leaf extracts (JRLE) in *P. berghei*-infected mice for the first time.

## Materials and methods

### Plant collection and commercial drug

Leaves of *Juglans regia* (walnut) were collected from Al Bahah City, Saudi Arabia. Authentication of the plant with voucher number KSU-21595 was carried out in the herbarium of the Botany Department, College of Science, King Saud University. Chloroquine diphosphate salt (Sigma-Aldrich, St. Louis, United States) was used as a commercially available standard antimalarial drug.

### Preparation of *Juglans regia* leaf extracts

The leaves of *J. regia* were washed with water, shade-dried, and coarsely powdered in a grinder. Walnut leaf extracts were prepared according to the method described by Manikandan et al. (36). In brief, the air-dried powder (100 g) of walnut leaves was extracted by percolation with 70% methanol for higher extraction efficiency and kept in the refrigerator for 24 h, filtered, and concentrated under reduced pressure in a rotatory evaporator at 55°C. To evaporate the alcohol and obtain a dry extract, it was placed at 37°C for 15 h. The residue was collected and placed in sealed bottles. The freeze-dried extracts were kept at −20°C until used for antimalarial testing.

### Fourier-transform infrared spectroscopy

A small quantity of JRLE was mixed with potassium bromide as 1: 99 wt % and ground to form a uniform consistency, then pulverized and analyzed using Thermo Scientific’s optical spectrometer NICOLET 6700 (Waltham, United States), according to the recommended protocol of Negrea et al. (37). Maximum absorption was reported in the number of waves in a range of 4,000–400 cm^−1^ at 25°C.

### Activation of *Plasmodium* parasite

Cryopreserved *P. berghei* parasite was passaged four times in laboratory mice (*Mus musculus*, *n* = 5). Parasitemia of infected mice was measured by staining thin tail blood smears with 10% Giemsa solution (Sigma-Aldrich; diluted in PBS), using the method of Hilou et al. (38). Parasitemia (% of pRBCs) was evaluated by microscopic count and calculated as follows: [(number of pRBCs)/(total numbers of RBCs counted)] × 100. When parasitemia reached about 20%, parasitized blood was collected in a heparinized tube and diluted in 200 μL of phosphate-buffered saline (PBS; pH 7.4) to 1 × 10^5^ parasitized erythrocytes/ml (39). The infection of C57BL/6 female mice (experimental animals) was initiated by the passage of the *P. berghei* parasite via an intraperitoneal injection (40). Each mouse received 100 μL of dilute infected whole blood containing 1 × 10^5^ parasitized erythrocytes.

### Experimental animals

Female mice of the C57BL/6 strain (*n* = 30) were used in this study. Animals were obtained from the animal facility in the College of Pharmacy (King Saud University, Saudi Arabia). Mice were approximately 9–12 weeks old at the start of the study (weights in the 20–25 g). They were housed in groups of 5 mice per polypropylene cage for 7 days before being used in the experiment. Mice were maintained at room temperature (23°C ± 2°C) in a 12 h light/dark cycle, given free access to standard laboratory animal food and water *ad libitum*. This study was approved by the Research Ethics Committee (REC) for Laboratory Animal Care at King Saud University (KSU-SE-24-74).

### Experimental design

Experimental mice were divided into six groups of five each, as follows:

Group 1: Non-infected and non-treated (negative control).

Group 2: Non-infected and treated with the potentially effective JRLE dose with low parasitemia (%).

Group 3: Infected and non-treated (positive control).

Group 4: Infected and treated with JRLE at 250 mg/kg of body weight (B.W.).

Group 5: Infected and treated with JRLE at 500 mg/kg of B.W.

Group 6: Infected and treated with the antimalarial drug, chloroquine (CQ), at 10 mg/kg of B.W.

All groups, except groups (1) and (2), were intraperitoneally injected with 1 × 10^5^ *P. berghei*-parasitized erythrocytes, according to Timms et al. (41). The mice were then left for 3 days post-infection. On day 4, treatments were administered orally as follows: groups (4 and 5) were orally treated with different concentrations of JRLE, according to Sharif et al. (42), and group (6) was treated with chloroquine, according to Abay et al. (43). Also, each mouse was weighed on day 9 and the difference between the pre- (day 1) and post-infection with *P. berghei* (day 9) body weights was calculated according to Dkhil et al. (44). On day 9 p.i., all mice were sacrificed by CO_2_ asphyxiation, dissected, and then samples were obtained.

### Parasitemia and survival rates

From the 3^rd^-day post-infection (dpi) onwards, the parasitemia of each infected experimental mouse was measured by thin blood smear, as earlier described. Parasitemia (%) was determined as described above. *P. berghei*-infected, infected-JRLE, and infected-CQ mice were monitored daily, and the time of death (days) was promptly registered to determine the survival rate curve, according to Ezike et al. (45). Each group’s survival time was calculated using the following equation: [total survival time of animals in the group/number of animals in the group].

### Sample collection

After the scarification of mice on day 9, the whole brain was removed from the mouse skull. Brain index was calculated using the equation: [(Brain weight.gm/mouse weight) × 100]. The brain was divided into two halves along the midline, according to Al-Shaebi et al. (39), and then preserved: (i) In neutral buffered formalin (NBF), it was used for histopathological examination. (ii) it was wrapped and stored at −80°C for neurotransmitter estimation in clean plastic films. (iii) In RNA later, it was stored at −80°C for later use in gene expression assay.

### Histopathological examination

According to Ogundolie et al. (46), the fixed brains in 10% NBF were used for histopathology analysis. The tissues were dehydrated in graded ethyl alcohol, treated with xylene, socked in paraffin wax, and finally cut into 5 μm thickness using a microtome machine. These sections were stained with hematoxylin and eosin (H&E) using standard protocols. Sections were examined and photographed using an Olympus B × 61 microscope (Tokyo, Japan).

### Immunohistochemical study

Detection of Glial Fibrillary Acidic Protein (GFAP) was performed according to Varma et al. (47). Brain sections (5 μm) were deparaffinized as per standard protocol. Sections were rehydrated in descending alcohol series. Antigen retrieval of all slides was done, after which each slide was treated with methanol containing 4% H_2_O_2_ for 30 min followed by placement for 10 min in 0.05 M Tris–HCl buffer (pH 7.4). Sections were covered with the primary antibody of mouse anti-GFAP (BioGenex, United States) in a 1:200 dilution in phosphate-buffered saline (PBS) and incubated for 45 min in a humid chamber at room temperature. Following removal of the primary antibodies and repetitive rinsing in PBS, sections were incubated with a biotin-conjugated secondary antibody of anti-mouse antiglobulin in PBS (Santa Cruz Biotechnology) containing carrier protein and sodium azide (15 mmol/L) large volume (Universal BioGenex kit). Horseradish peroxidase (HRP) conjugated streptavidin was used to cover the brain sections at room temperature and incubated for 30 min. After rinsing, slides were covered with substrate chromogen solution and incubated at room temperature until the optimum brown color peroxidase product was developed. Counter staining was done with hematoxylin (Sigma Chemical Co.). All sections were photographed using an Olympus B × 61 microscope (Tokyo, Japan).

### Neurotransmitters estimation

Contents of epinephrine (E), norepinephrine (NE), dopamine (DA), and serotonin (5-HT) were determined in the isolated brain samples, according to the method of Ciarlone (48). In brief, 0.3 mL of tissue homogenate was combined with 0.1 N hydrochloric acid and 3 mL of butanol to prepare duplicate internal standard tubes for neurotransmitter analysis. After centrifugation at 1,000 × g for 5 min, 2.5 mL of the supernatant was mixed with 1.6 mL of 0.1 N hydrochloric acid and 5 mL of heptane, vortexed for 2 min, and re-centrifuged. The organic phase was discarded, with 0.2 mL of the aqueous phase reserved for 5-HT analysis and 1 mL for E, NE, and DA analysis, kept on ice. For 5-HT external standards, 0.2 mL samples in 0.1 N hydrochloric acid had 1.2 mL of o-Phthalaldehyde added, mixed, and heated in a water bath for 10 min before spectrophotometric reading at 470 nm. For E, NE, and DA, 300 mg of neutral alumina was added, followed by shaking and centrifugation. The supernatant was discarded, and 4 mL of distilled water was added, followed by elution with 1.5 mL of 0.2 N acetic acid. External standards for E, NE, and DA were made in 0.2 N acetic acid with 0.2 mL EDTA added. A reagent blank was prepared with alkaline sulfite, iodine, and acetic acid. After heating, fluorescence was measured for E and NE at 480 nm and DA at 375 nm.

### Quantitative real-time polymerase chain reaction

RNA was extracted from the brain tissues tissue using the Qiagen RNeasy Plus Mini kit. Concentration and purity for each RNA sample were detected by NanoDrop ND-1000 Spectrophotometer (NanoDrop Technologies, Wilmington, Delaware, United States) using 260 and 260/280 nm ratios. The RNA samples were reverse-transcribed using a high-capacity RNA-to-cDNA Master Mix kit to prepare cDNA. The primers used in the present study were designed using Primer-BLAST, and amplification was done using the specific PCR primers for different genes involved in neurotransmitter signaling and Thermo Scientific Maxima® SYBR Green/ROXqPCR Master Mix (2×) by Rotor-Gene Q (Qiagen, United States) (Table 1). The resulting Threshold Cycle (Ct) value determined the 2^−ΔΔCt^ of mRNA expression of the different genes (49). The reference gene was beta-actin (*β*-actin).

**Table 1:** Primers used for real-time PCR analysis of genes involved in neurotransmitter signaling.

Genes	Primer direction	Primer sequence (5′ → 3′)
Upregulated genes during infection		
Cholinergic signaling		
Cholinergic receptor, nicotinic, β polypeptide 2 (Chrnb2)	Forward	5′-ACTCCTCCCCTAGTAGTTCCAC-3′
	Reverse	5′-CAAAGGAGGCCAAAGCTGAAC-3′
GABAergic signaling		
Gamma-aminobutyric acid B receptor 1 (Gabbr1)	Forward	5′-GGAAGGAGAGACGGGGGT-3′
	Reverse	5′-CACAGGAATCCAGGCTCCAG-3′
G-proteins		
Guanine nucleotide-binding protein α inhibiting 1 (Gnai1)	Forward	5′-GCGGGAGCTGAGGACGTAG-3′
	Reverse	5′-TTGCGGTCGATCATCTTGCTG-3′
Downregulated genes during infection		
Glutamatergic signaling		
Glutamate ionotropic receptor AMPA type subunit 2 (Gria2)	Forward	5′-CGGGAGGGTGCTGATATTCC-3′
	Reverse	5′-CCCTCGTTTCCCTTTCCTCC-3′
Reference gene		
Beta-actin (β-actin)	Forward	5′-AGGGAAATCGTGCGTGACAT3′
	Reverse	5′-GGAAAAGAGCCTCAGGGCAT-3′

### Sandwich enzyme-linked immunosorbent assay

Using mouse enzyme-linked immunosorbent assay (ELISA) kits, the level of neurotransmitter markers, including Gamma-Aminobutyric Acid (GABA) (Cat. MBS269152, MyBioSource, United States) and Glutamate (Cat. KA1909, Abnova, United States), was investigated following the protocol instructions. Optical densities (OD) of outcomes from the brain samples were measured using the Bio-Rad IMark Microplate Reader SW 1.04.02.E. Based on a standard curve, OD values were converted to concentrations and presented as ng/mg (for GABA) and ug/mg (for glutamate).

### Statistical analysis

The data from individual experiments was presented as mean ± SD. Differences between groups were analyzed using one-way analysis of variance (ANOVA) through the SPSS v.18 software program (SPSS Inc., Chicago, Illinois, United States). The minimum criterion for statistical significance was set at a *p*-value ≤ 0.05 for all group comparisons.

## Results

**Figure 1:**
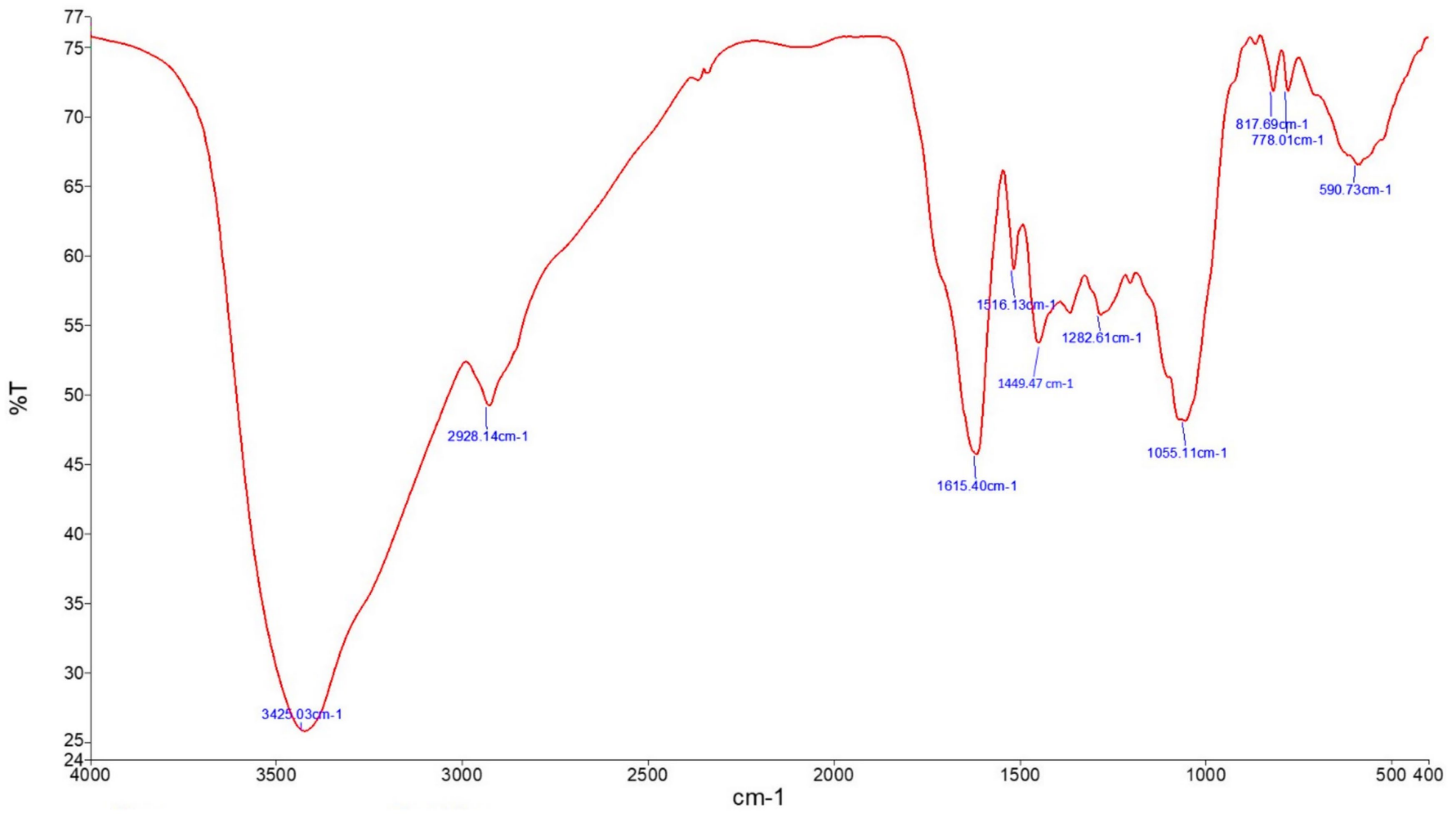
FT-IR of *Juglans regia* leaf extracts in an aqueous medium showing the functional characteristic of the material.

The analysis of JRLE using FT-IR showed major bands at 3,425.03, 2,928.14, 1,615.40, 1,516.13, 1,449.47, 1,282.61, 1,055.11, 817.69, 778.01, and 590.73 cm^−1^ (Figure 1). O-H stretching was indicated by the band at 3,425.03 cm^−1^ confirming the presence of alcohol. The band at 2,928.14 cm^−1^ implied C-H stretching for the presence of alkane. C=C stretching at 1,615.40 cm^−1^ confirming the presence of *α*,*β*-unsaturated ketone. The band at 1,516.13 cm^−1^ implied N-O stretching for the presence of nitro compound. C-H stretching at the band 1,449.47 cm^−1^ confirmed the presence of alkane. The band 1,282.61 cm^−1^ (C-O stretching), 1,055.11 cm^−1^ (C-O stretching), 817.69 cm^−1^ (C=C stretching), and 778.01 cm^−1^ (C-H stretching), 590.73 cm^−1^ (C-I stretching) confirmed the presence of aromatic ester, primary alcohol, alkene, 1,2,3-trisubstituted, and halo compound, respectively (Table 2).

**Table 2:** FT-IR for *Juglans regia* leaves extract.

Absorption (cm^−1^)	Transmittance (%)	Appearance	Group	Compound class
3,425.03	4.698467	Strong, broad	O-H stretching	Alcohol
2,928.14	8.986099	Medium	C-H stretching	Alkane
1,615.40	8.340388	Strong	C=C stretching	α,β-unsaturated ketone
1,516.13	10.78168	Strong	N-O stretching	Nitro compound
1,449.47	9.81128	Medium	C-H bending	Alkane
1,282.61	10.17991	Strong	C-O stretching	Aromatic ester
1,055.11	8.782223	Strong	C-O stretching	Primary alcohol
817.69	13.12944	Medium	C=C bending	Alkene
778.01	13.12981	Strong	C-H bending	1,2,3-trisubstituted
590.73	12.16071	Strong	C-I stretching	Halo compound

**Figure 2:**
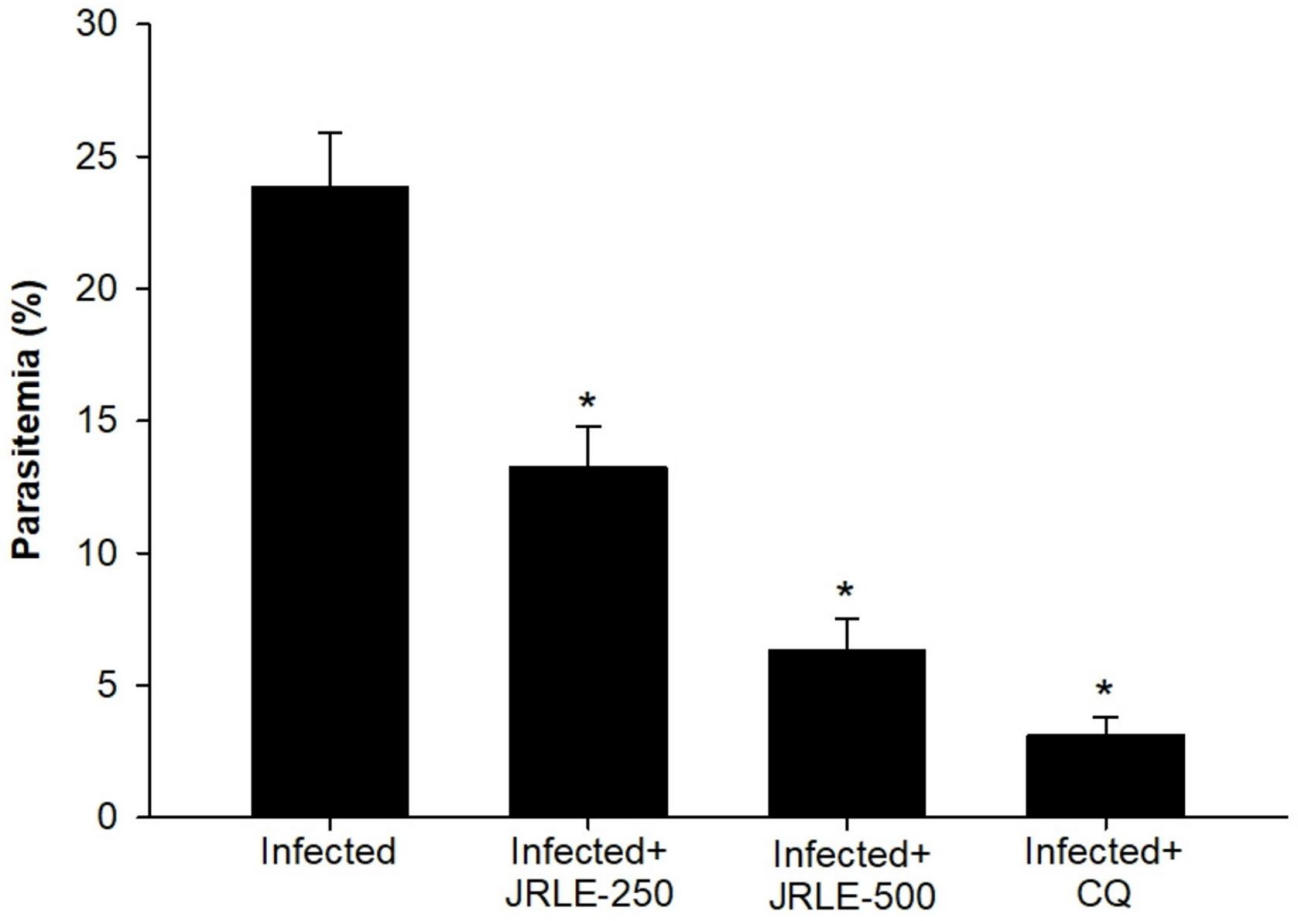
Percentage of parasitemia in mice infected with *Plasmodium berghei* and treated with various doses of JRLE or chloroquine (CQ). Data are presented as mean ± SD at *p* ≤ 0.001. ^*^Significance against the infected group.

Parasitemia levels were assessed using Giemsa-stained blood smears (Figure 2). On day 9 post-infection, mice infected with *P. berghei* at an inoculum of 1 × 10^⁵^ parasitized erythrocytes exhibited a parasitemia level of 23.84% ± 2.06%. Treatment with plant extracts significantly reduced parasitemia in a dose-dependent manner. Specifically, doses of 250 mg/kg and 500 mg/kg of JRLE reduced parasitemia to 13.25% ± 1.53% and 6.33% ± 1.18%, respectively. In contrast, infected mice treated with chloroquine exhibited the lowest parasitemia level (3.11% ± 0.69%), demonstrating that the standard drug exhibited the highest antiplasmodial activity compared to JRLE.

**Figure 3:**
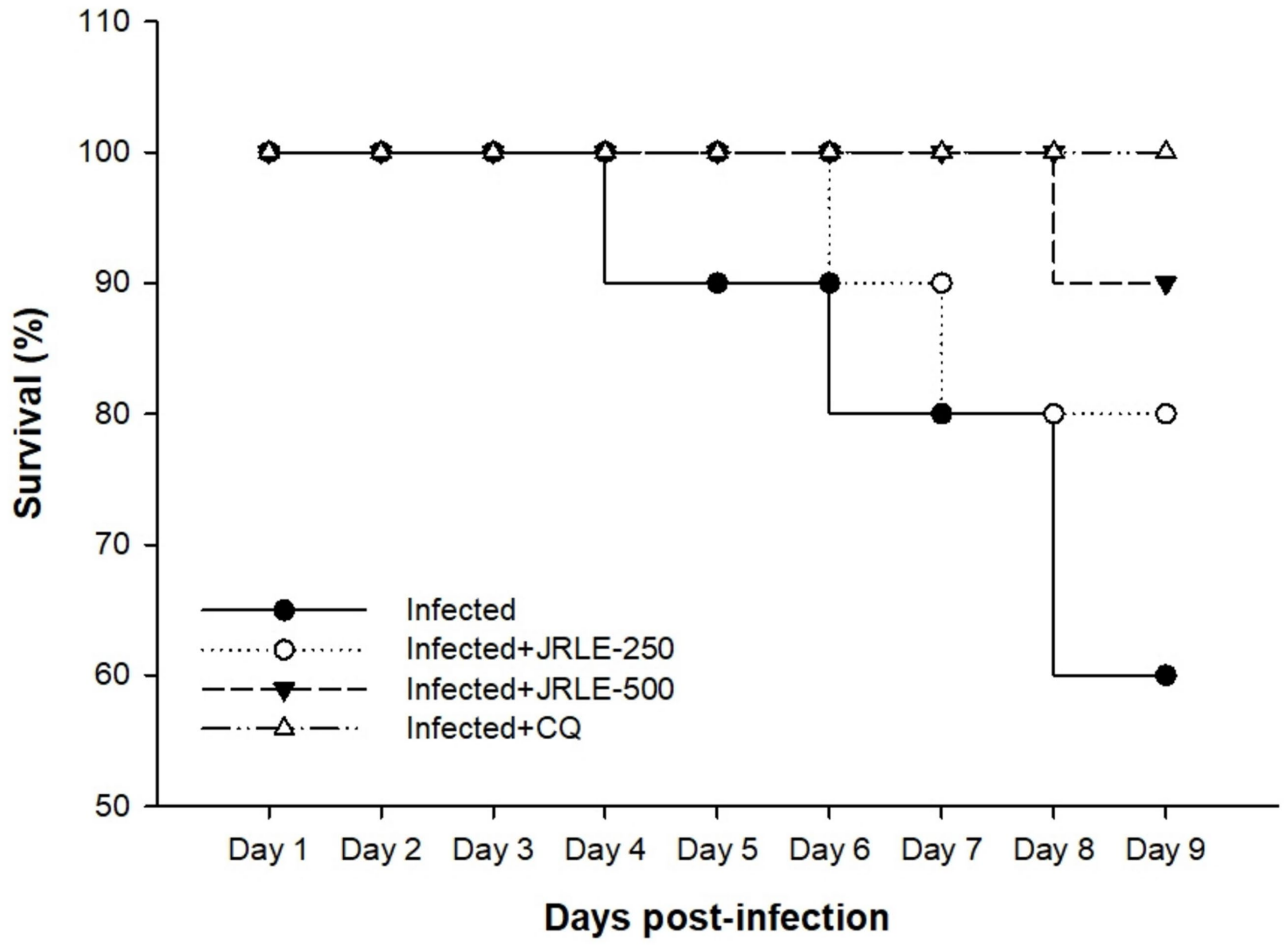
Survival percentage of the infected and treated mice groups during experimental study.

Figure 3 shows the survival rate of experimental animals in each group concerning the time of infection. Our data showed that during *P. berghei* infection, mice in the infected group started to die by day 3 p.i. Treatment with JRLE in a dose of 250 mg/kg affected the mortality rate of *P. berghei*-infected mice, which began to die by day 7 p.i. On the other hand, treatment with 500 mg/kg JRLE improved the clinical outcomes of *Plasmodium* infection and expanded the survival time until day 9 p.i. (Figure 3). All the mice in the CQ group survived during the experiment (Figure 3).

**Figure 4:**
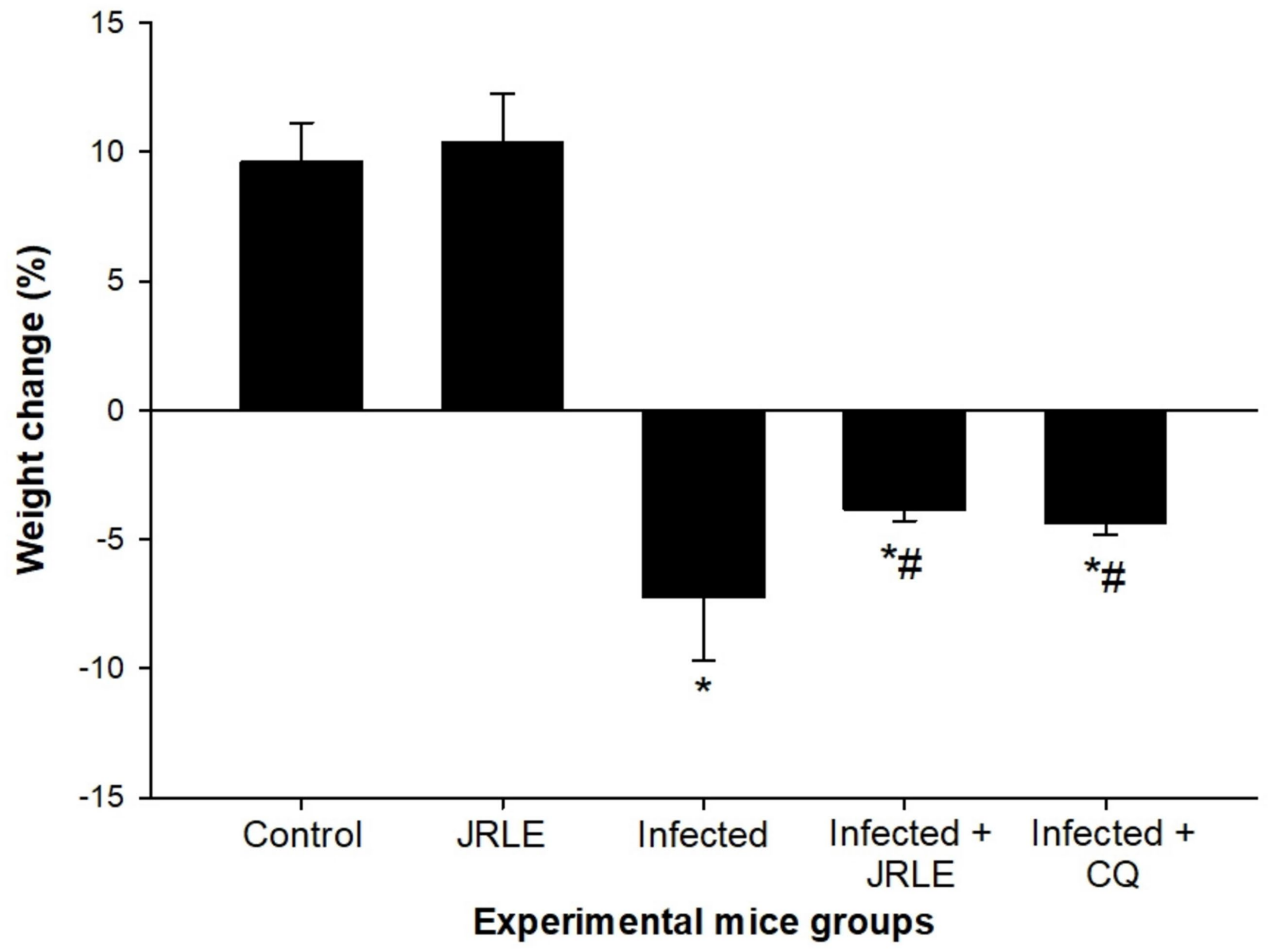
JRLE improved weight loss due to infection with *Plasmodium berghei* in mice. Values are means ± SEM. ^*^Significance (*p* ≤ 0.05) against non-infected control group, ^#^Significance (*p* ≤ 0.05) against infected group.

The mean body weight (BW) analysis over 9 days is illustrated in Figure 4, highlighting significant differences between the studied groups. On day 9 post-infection (p.i.), the control group exhibited a significant BW increase of 9.62%. In contrast, *P. berghei* infection caused severe weight loss in the infected group, with an average decrease of −7.25%. Treatment with JRLE or chloroquine (CQ) significantly mitigated the weight loss. The JRLE-treated group showed an average BW change of −3.82%, while the CQ-treated group had a slightly greater reduction in BW, averaging −4.34%. These results indicate that both treatments improved weight retention, although chloroquine had a slightly lower effect compared to JRLE.

**Figure 5:**
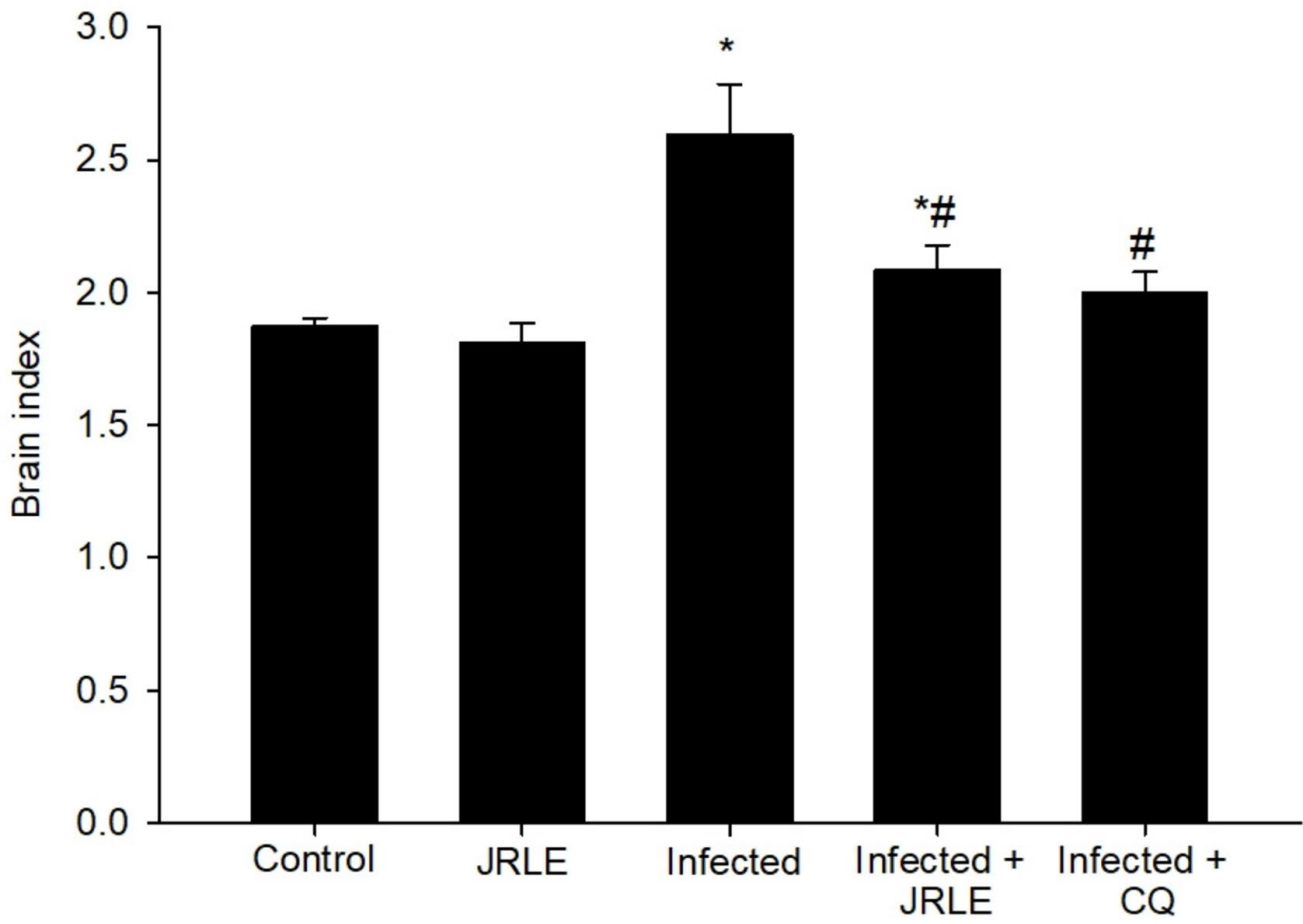
Brain index among the experimental mice groups. Values are means ± SEM. ^*^Significance (*p* ≤ 0.05) against non-infected control group, ^#^Significance (*p* ≤ 0.05) against infected group.

The brain index, calculated as the ratio of brain weight to body weight, revealed significant differences among the groups of infected mice. The infected group exhibited a notably higher brain weight compared to the control group (Figure 5). By day 9 post-infection (p.i.), the infected mice showed a significant increase in brain weight, averaging 2.59 ± 0.19. Treatment with JRLE effectively reduced the brain weight to 2.08 ± 0.09, closely resembling the brain weight observed in the chloroquine-treated group (2.00 ± 0.07) (Figure 5). These results highlight the potential of JRLE in mitigating the effects of *P. berghei* infection on brain weight.

**Figure 6:**
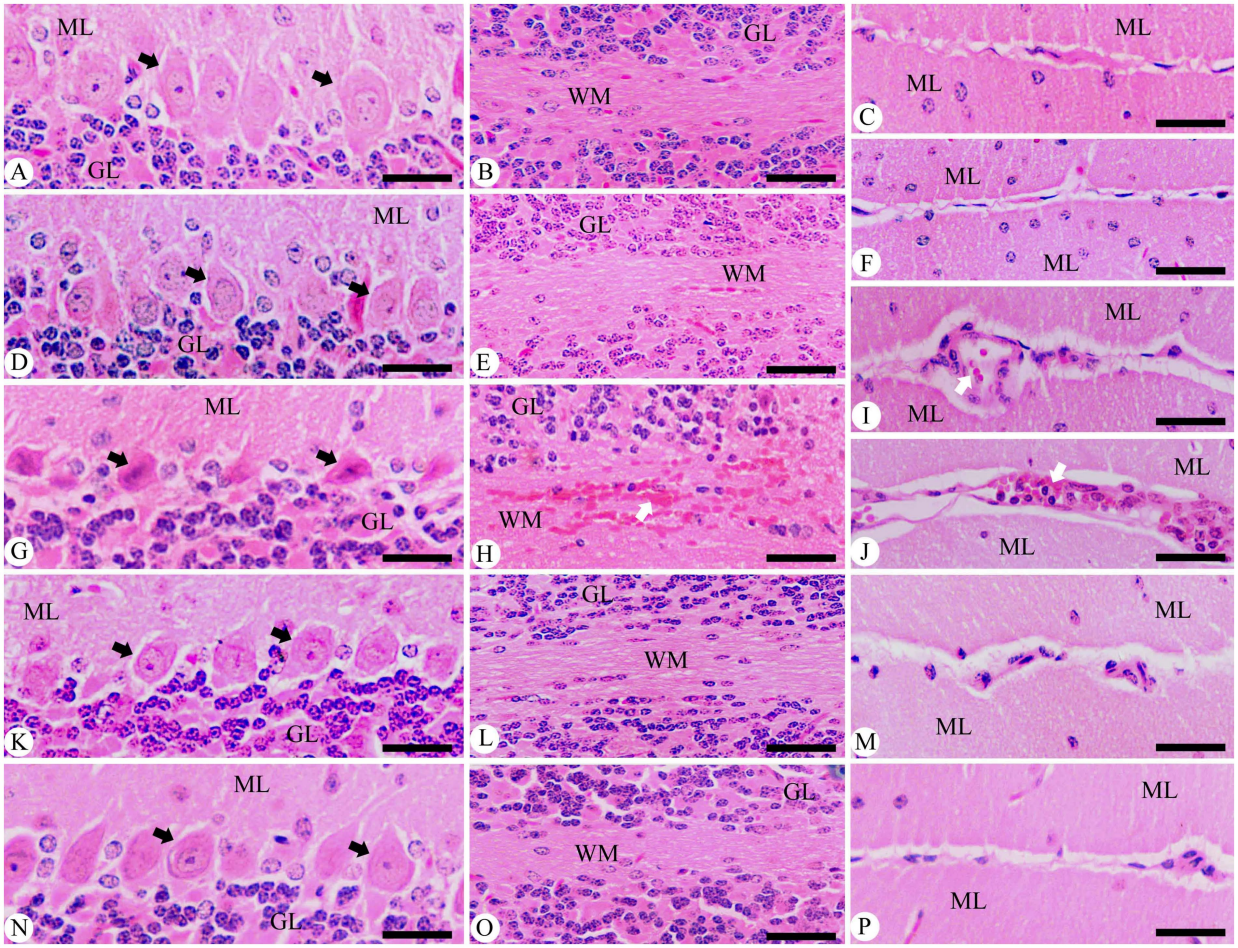
Photomicrographs of the cerebellum in different experimental groups on day 9 p.i. with *Plasmodium berghei*. **(A–C)** Control group. **(D-F)** non-infected-treated group with 500 mg/kg of JRLE. **(G–J)** Infected group. **(K–M)** Infected-treated group with 500 mg/kg of JRLE. **(N–P)** Infected-treated group with chloroquine. ML, molecular layer; GL, granular layer; WM, white matter; Black arrows, Purkinje cell; White arrows, hemorrhage. Sections were stained with hematoxylin and eosin. Scale Bar = 100 μm.

To better understand the impact of *P. berghei* infection on brain status, hematoxylin–eosin staining sections were utilized to characterize histopathological changes in the cerebellum (Figure 6). The findings show that brain tissue sections from control mice, as well as those in the non-infected treated group with 500 mg/kg, exhibited no morphological alterations, which is reassuring. In contrast, the *P. berghei*-infected animals revealed concerning progressive morphological changes by day 9 p.i. These included degeneration of Purkinje cells, cerebral hemorrhage in dilated sinusoids, intravascular sequestrations of parasitized red blood cells (pRBCs), and lymphocyte infiltration (Figure 6). Notably, the histological analysis of brain tissue from infected mice treated with 500 mg/kg JRLE showed significant improvements, resembling the histological features of the chloroquine-treated group (Figure 6).

**Figure 7:**
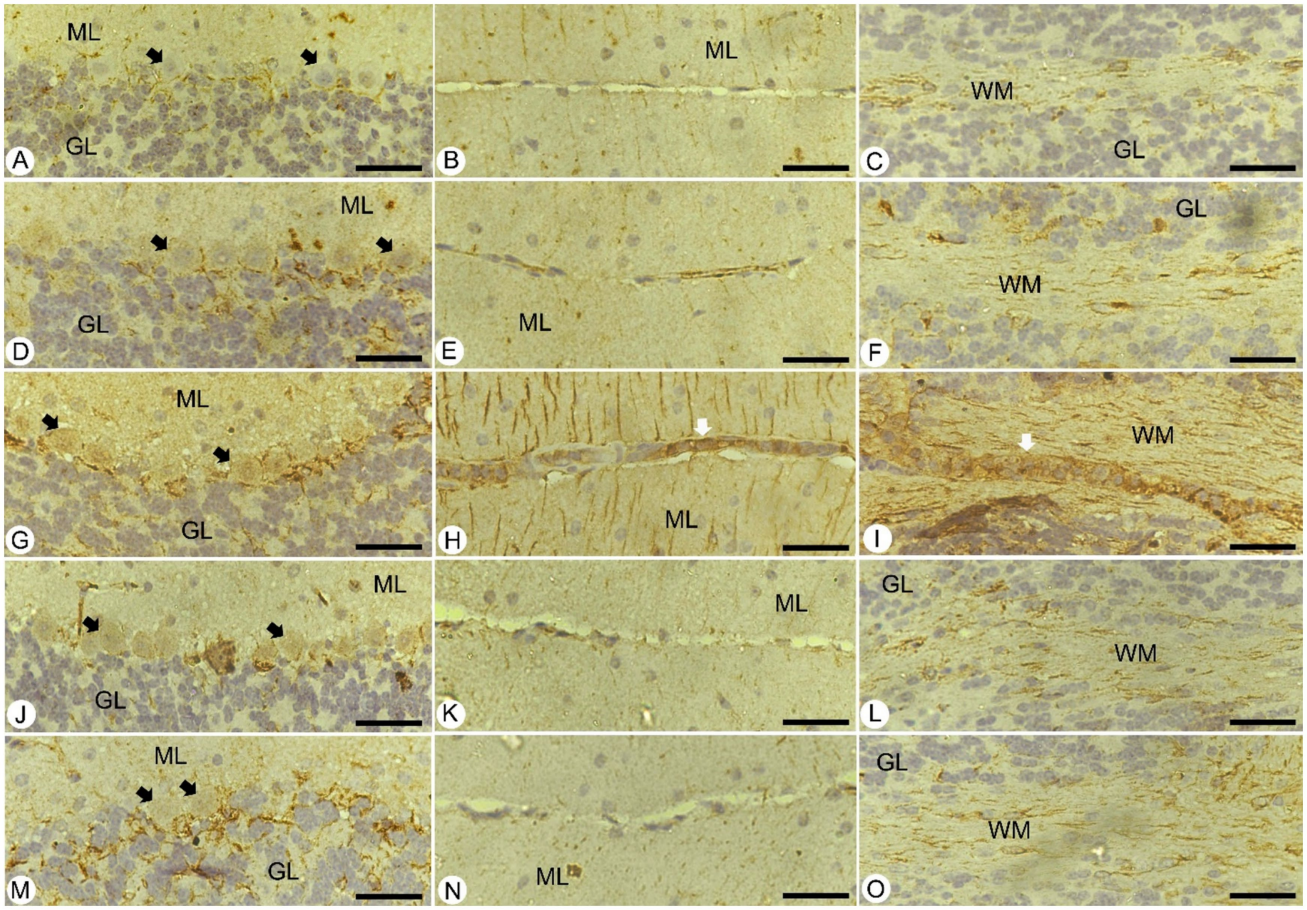
Immunohistochemical localization of GFAP in the brain of mice. **(A–C)** Control group. **(D–F)** non-infected-treated group with 500 mg/kg of JRLE. **(G–I)** Infected group. **(J–L)** Infected-treated group with 500 mg/kg of JRLE. **(M–O)** Infected-treated group with chloroquine. ML, molecular layer; GL, granular layer; WM, white matter; Black arrows, Purkinje cell; White arrows, hemorrhage. Scale Bar = 100 μm.

For a deeper understanding, brain sections from different experimental groups underwent immunohistochemical staining to assess GFAP-positive cells in the cerebellum. These cells play a critical role in maintaining structural stability and integrity, alongside supporting the homeostatic functions of astrocytes (Figure 7). In the control group, the presence of GFAP-positive cells was within normal limits. Unfortunately, *Plasmodium* infection led to an increase in these cells around the Purkinje cell layer and in both the cerebellar molecular and white layers compared to normal levels in control mice (Figure 7). Notably, these changes were more pronounced in areas of neuronal loss. Following treatment with JRLE, GFAP expression significantly decreased in the brains of mice affected by *P. berghei*, indicating a reduction in cellular reactivity compared to the infected group.

**Figure 8:**
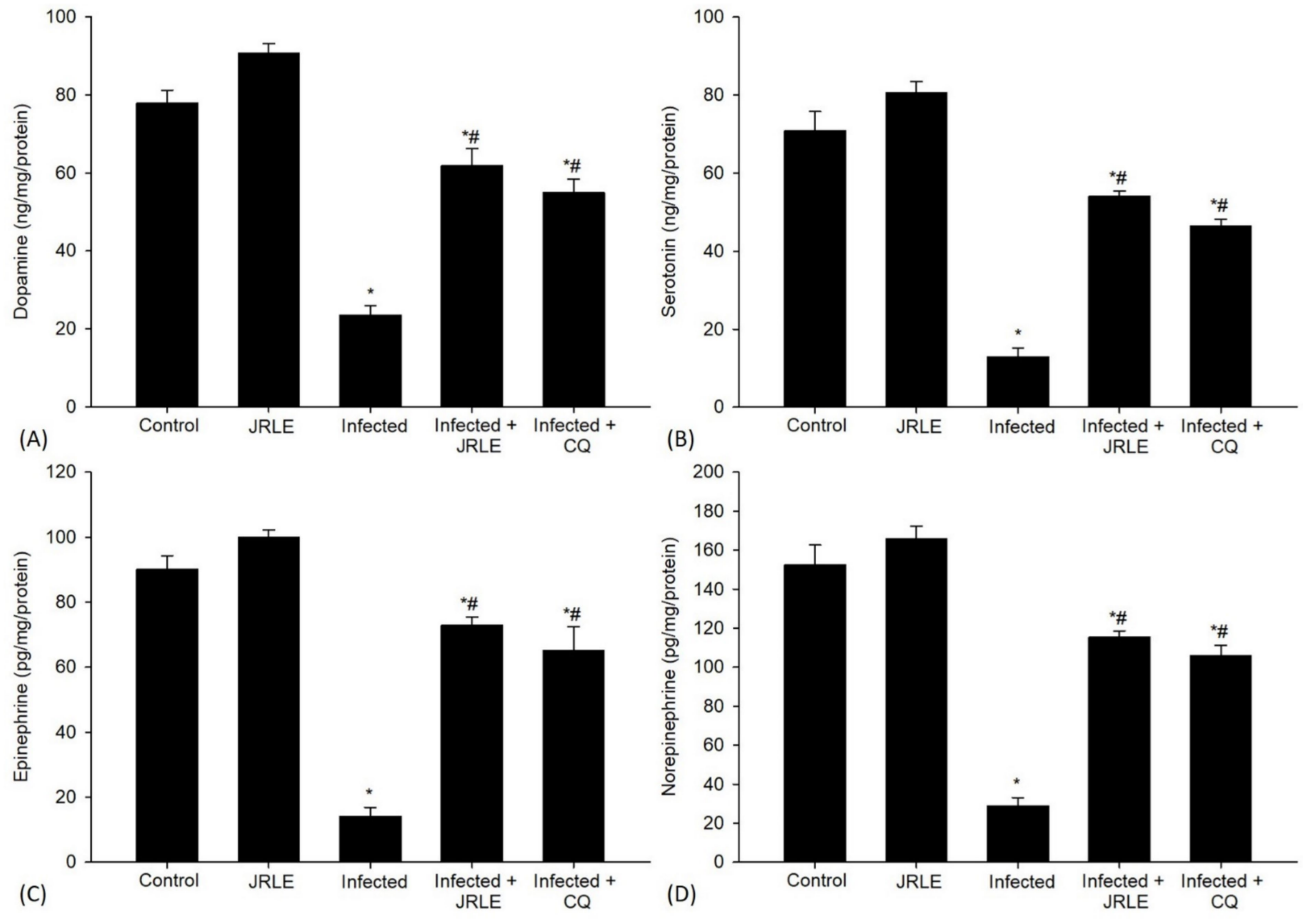
Neurotransmitters in the brain samples from different experimental mice groups in the 9^th^ day p.i. **(A)** Dopamine. **(B)** Serotonin. **(C)** Epinephrine. **(D)** Norepinephrine. ^*^Significant change concerning the control group, ^#^Significance change concerning the infected group.

A concerning aspect of the infection was the significant decrease in the levels of neurotransmitters like epinephrine (14.15 ± 2.74 ng/mg/protein), norepinephrine (28.93 ± 4.11 ng/mg/protein), dopamine (23.48 ± 2.50 ng/mg/protein), and serotonin (12.90 ± 2.23 ng/mg/protein) in the brains of the infected mice when compared to the control group (Figure 8). Upon treatment with JRLE, there was a notable increase in the neurotransmitter levels (72.95 ± 2.47, 115.43 ± 3.12, 61.82 ± 4.38, and 54.05 ± 1.38 ng/mg/protein, respectively) in the brain of infected mice compared to those that were merely infected (Figure 8).

**Figure 9:**
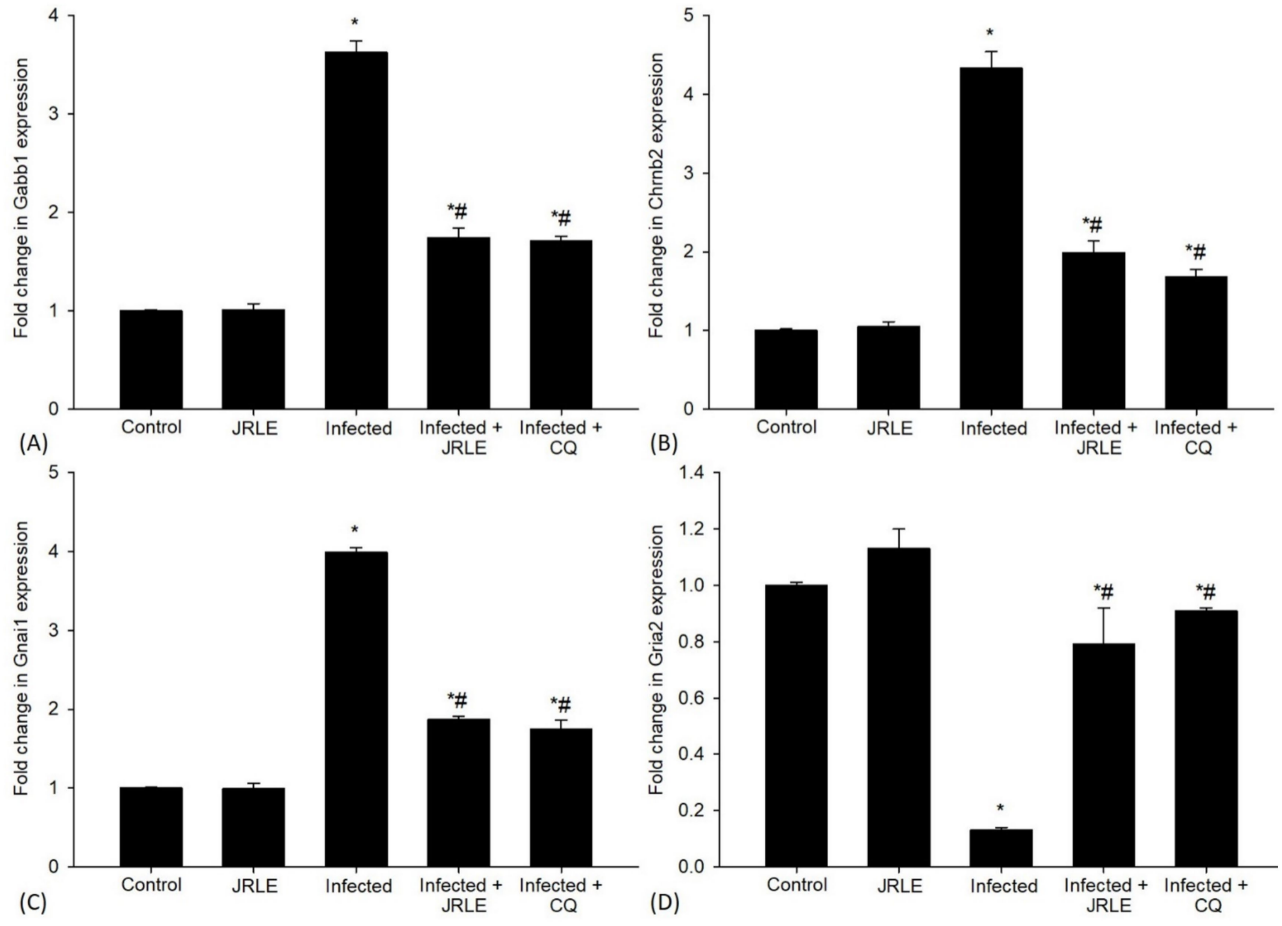
Effect of JRLE on the mRNA expression of some selected genes in the brain samples from different experimental mice groups in the 9^th^-day p.i. **(A)** Gabb1. **(B)** Chrnb2. **(C)** Gnai1. **(D)** Gria2. ^*^Significant change concerning the control group, ^#^Significance change concerning the infected group.

Quantitative RT-PCR (qRT-PCR) was utilized to examine changes in the mRNA expression levels of neurotransmitter signaling genes in the mouse brain (Figures 9A–D). The *P. berghei* infection led to a significant upregulation of the Gabbr1 gene expression, approximately 3.63-fold higher than the control group (Figure 9A). Treatment with JRLE brought a significant downregulation of this gene by about 1.74-fold, closely resembling the reference drug (1.71-fold) (Figure 9A). Moreover, *P. berghei* infection resulted in a significant upregulation of the nicotinic acetylcholine receptor subunit Chrnb2 (Figure 9B), with a 4.33-fold increase compared to the control group (Figure 9B). JRLE treatment showed significant downregulation of 1.99-fold for this receptor compared to the reference drug’s 1.68-fold (Figure 9B). The study also documented upregulation of the Gnai1 gene after infection, with an elevation of about 3.99-fold compared to the control group (1.00-fold) (Figure 9C). Treatment with JRLE significantly downregulated this gene by about 1.87-fold in comparison to the reference drug (1.75-fold) (Figure 9C). The infection led to a significant downregulation of the Gria2 gene expression to about 0.13-fold, but there was significant improvement after JRLE treatment, recording an upregulation of 0.79-fold compared to the chloroquine group (0.91-fold) (Figure 9D).

**Figure 10:**
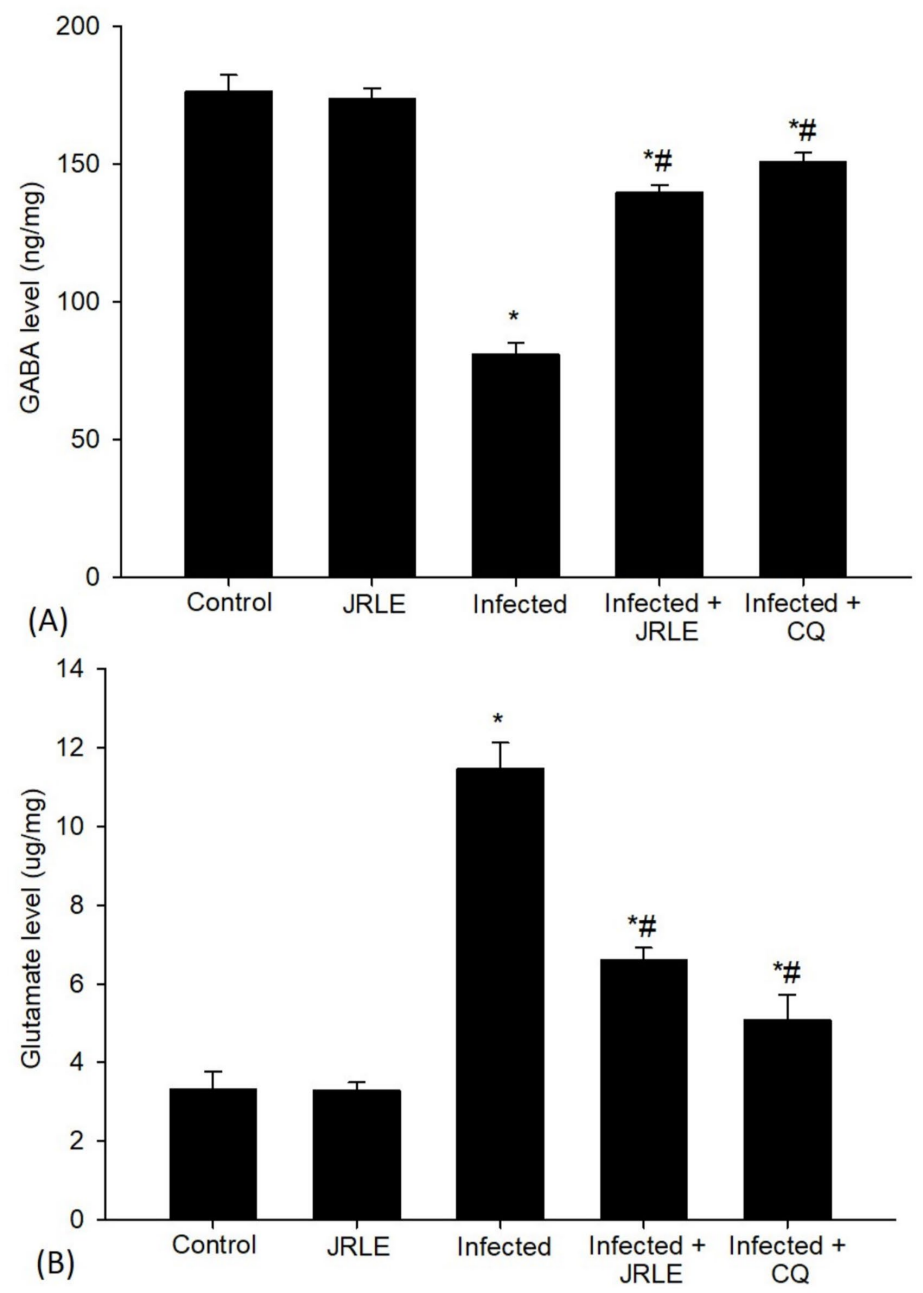
Levels of neurotransmitters of **(A)** GABA and **(B)** Glutamate in the brain samples from the different experimental mice groups. ^*^Significant change (*p* ≤ 0.05) concerning the control group, ^#^Significance change (*p* ≤ 0.05) concerning the infected group with *Plasmodium berghei*.

In our ongoing efforts to understand the effects of JRLE, we also investigated levels of GABA and glutamate using ELISA (Figure 10A). The results striking; compared to the control group, *Plasmodium* infection resulted in a significant decrease in GABA level (80.89 ± 4.26 ng/mg). Yet, it was uplifting to observe that JRLE treatment significantly elevated the *P. berghei*-induced decrease in GABA level (139.77 ± 2.70 ng/mg) compared to the infected group. Additionally, glutamate levels increased dramatically after *Plasmodium* infection (11.46 ± 0.67 ug/mg), correlating with behavioral changes when compared to the control group. The infected-treated group that received 500 mg/kg JRLE displayed a significant decrease in glutamate levels (6.60 ± 0.31 ug/mg) compared to the *P. berghei*-infected mice (Figure 10B).

## Discussion

Malaria, caused by an apicomplexan protozoan parasite of the genus *Plasmodium*, is a major global health problem, particularly in tropical and subtropical regions (5). The emergence of drug-resistant strains for *Plasmodium* underscores the urgent need for novel therapeutic strategies (50, 51). Developing new antimalarial compounds from different sources, especially traditional medicinal plants, offers a promising approach to addressing parasite resistance. Therefore, the present *in vivo* study evaluated the antimalarial activity of *J. regia* leaf extracts (JRLE) against *P. berghei* infection.

Infection of the mouse strain, C57BL/6, with *P. berghei* affords an experimental model of cerebral malaria that shares some aspects with human cerebral malaria (52). In this study, the course of infection in the infected mice reached a peak level of infection (23.84% ± 2.06%) on day 9. This result agreed with Rajan et al. (53) and Badejo et al. (54) reported the peak parasitemia achieved on day 9 during *P. berghei* infection. This study showed that JRLE has a dose-dependent *in vivo* antimalarial effect against the *Plasmodium* parasite. When JRLE (500 mg/kg) was administrated, the parasitemia was significantly reduced to 6.33 ± 1.18%. These results might be due to the bioactive compounds of JRLE (i.e., alkaloids, flavonoids, phenolic compounds, and terpenoids) which might be implicated in antiplasmodial activity. Flavonoids inhibit the biosynthesis of fatty acids in the parasite metabolism which is required for the development of *Plasmodium* parasites during the erythrocytic phase, this is agreed with data obtained by Zhao et al. (55). Moreover, tannins in JRLE may play a role in inhibiting the *Plasmodium* protease enzyme, so that its growth and development are inhibited and prevent the invasion of new erythrocytes, consistent with Kaushik et al. (56). Similarly, Serakta et al. (57) reported the antiparasitic activity of the *J. regia* hydroalcoholic extract that significantly inhibits the growth of the promastigotes of *leishmania major*.

In this study, *P. berghei*-infected mice died showing typical signs of cerebral malaria (i.e., hemiplegia, ataxia, epilepsy, and blindness), which agreed with the previous studies by de Souza and Riley (58). The JRLE affected the blood and tissue stages of *P. berghei* significantly. The JRLE also prolonged the survival time of the treated mice which is an indication of significant antimalarial activity associated with the presence of some bioactive secondary metabolites in the plant leaf extracts. Flavonoids in JRLE could counter the redox imbalance associated with *Plasmodium* infection, increasing the mean survival time of the infected treated mice. As previously reported, plant’s bioactive principles possess antiparasitic activity (59).

After infection, the weight loss exhibited by animals following *P. berghei* infection was correlated with high parasitemia due to the disturbances in the mice’s metabolism and the loss of mice’s appetite that might have resulted from parasite proliferation, which agreed with previous studies (44, 60–65). Treatment with JRLE induced the body weight of the infected mice. This might be due to the presence of bioactive components (i.e., sterols in the plant extract) which could enhance nutrient absorption and aid in animal digestion, this is agreed with Thakur and Singh (66) and Rock et al. (67).

In the brains of the infected mice, there were some histopathological alterations in the cerebellum including degeneration of Purkinje cells, hemorrhagic damage, infiltration of lymphocytes, and intravascular sequestrations of pRBCs which is consistent with the wide-ranging patterns of reported neurocognitive sequelae after cerebral malaria infection. These results agreed with previous studies by Bopp et al. (68), Dkhil et al. (44), Al-Shaebi et al. (39), Mohanty et al. (69), and Coughlan et al. (70). According to Maung and Than (71), during *P. berghei* infection, extravasation of RBCs is not only due to the rupture of blood vessels but also an increase in vascular permeability which results in the release of RBCs, both uninfected RBCs and infected with trophozoite. After the *Plasmodium* infection, a significant elevation of the brain index was observed in the infected group. This may be due to the presence of brain edema during cerebral malaria (72). Upon treatment, the JRLE not only inhibited the growth of the *Plasmodium* parasite but also caused a decrease in the overall pathologic effect of the parasite in the mice through the improvement in the brain tissue of the infected mice. This data is consistent with Aydın et al. (73), Ansari et al. (74), Rusu et al. (75), and Sharma et al. (76). Moreover, Edenharder and Grünhage (77) and Salter et al. (78) reported that flavonoids in *J. regia* could protect cells by acting as free radical scavengers, inhibiting DNA damage and mutagenicity.

GFAP is a brain-specific protein that functions as the major integral component of the cytoskeleton of astrocytes (79). Although few gross histopathological changes were observed, a significant increase in overall GFAP staining was observed around the Purkinje cell layer and in both the cerebellar molecular and white layers, indicating the dysfunction of astrocytes after *Plasmodium* infection. This is agreed with Missler et al. (80) and Herbas et al. (81) reported that GFAP was significantly increased after the infection in C57BL/6 mice with *P. berghei* ANKA. According to Carter et al. (82), astrocytes are crucial in maintaining the blood–brain barrier and the neurovascular unit. Their dysfunction could release GFAP, explaining its elevations using immunohistochemistry. These results agreed with previous studies by Chen and Swanson (83), and Sofroniew (84) proposed that GFAP expression is a cardinal feature of many pathological conditions of the central nervous system (CNS) and astrocytes. Treatment with JRLE induced protection of the mice’s brain from cerebral injury by inhibiting GFAP expression. Similarly, to our study, they investigated a strong neuroprotective effect of JRLE against cognitive deficits (74, 76).

Several biomarkers associated with cerebral injury were examined in the *P. berghei*-infected mice. Neurotransmitters are chemicals found in nerve cells that facilitate communication between cells at synapses, playing a critical role in signaling regulation (85). Our results indicated that the brains of the infected mice exhibited reduced contents of neurotransmitters, specifically epinephrine, norepinephrine, dopamine, and serotonin. This finding is consistent with Zai et al. (86), who suggested that the GABA_B_ receptor plays a physiological role in modulating GABA release through an autoreceptor, as well as influencing the release of other neurotransmitters such as noradrenaline (87) and serotonin (88), cholecystokinin (89), and Somatostatin (90). The decrease in neurotransmitter levels may contribute to elevated body temperature, increasing malaria risk (91). Oyewole et al. (92) reported an increase in serotonin levels in the brains of infected mice following the lysis of platelet cells. Generally, some parasitic infections, such as *Toxoplasma gondii* (93), *Schistosoma mansoni* (94), *Toxocara canis* and *Trichinella spiralis* (95), lead to alterations in neurotransmitter levels. In our study, oral treatment with JRLE significantly increased neurotransmitter levels. This may be attributed to the role of juglone, as stated by Thakur (96) and Cintesun et al. (97).

Regarding gene expression, cerebral malaria is fundamentally a neurological disease, as evidenced by substantial changes in gene expression in the brain (98). Several gene expressions recorded in this study included Chrnb2, Gabbr1, Gnai1, and Gria2, all showing significant alterations compared to those in the control mice. This aligns with findings from Desruisseaux et al. (98) and Mubaraki et al. (99), who reported changes in many encoding proteins in mice infected with *P. berghei* ANKA that exhibited neuropathology. The Chrnb2 gene encodes a protein that forms a subunit of a larger protein known as a neuronal nicotinic acetylcholine receptor (nAChR), which is part of a superfamily of ligand-gated ion channels and is crucial for fast transmission at synapses (100). Our findings demonstrated high expression levels of the Chrnb2 gene associated with damaged Purkinje cells in the *P. berghei*-infected mice group compared to the control group. Additionally, the Gria2 gene, which is part of the family of glutamate receptors sensitive to alpha-amino-3-hydroxy-5-methyl-4-isoxazole propionate (AMPA), functions as ligand-activated cation channels (101). However, Gria2 expression was lower in *P. berghei*-infected mice compared to the control group. Given the physiologic significance of the GABA_B_ receptor in neurobiology, Gabbr1 is considered an attractive candidate gene for neuro-behavioral disorders (86). The Gabbr1 gene was found to be significantly over-expressed in the infected group that had been induced by cerebral malaria. According to Priya et al. (102), there is a close relationship between neuronal activity and energy metabolism. In this study, the G-proteins inhibitory *α* subunits represented by Gnai1 were overexpressed at 9 days post-infection (p.i.) in the brain of infected mice. Narzi et al. (103) noted that Gnai1 is associated with an inhibition mechanism of ATP-bound adenylyl cyclase type 5. The upregulation of Gnai1 was linked to decreased relapse-free survival, consistent with Li et al. (104). Generally, JRLE treatment significantly reduced the expression of Chrnb2, Gabbr1, and Gnai1 genes in the infected mice except for the elevation of Gria2. This finding highlights the ability of JRLE to relieve the burden of malaria pathogenesis suggesting that this plant extract may have neuroprotective properties as noted by Ansari et al. (74), Liu et al. (105), Sharma et al. (76), and Yeh et al. (106).

Our results indicated that the brains of the infected mice had lower levels of GABA. This reduction may be due to the *Plasmodium* parasite, which affects the action of GABA by inhibiting the permeability of nerve cell membranes to chloride ions (Cl^−^) from the extracellular environment. This process leads to membrane depolarization and results in physiological disruption. This data supports the findings of Kantrowitz et al. (107) and Heuer and Grosell (108), which indicated that dysfunction in the GABAergic system and the resulting reduction in GABA’s inhibitory effects can lead to various pathological processes and the development of diseases. After treatment, the active constituents of the plant extract play a significant role in mitigating GABA’s effects and restoring its function in neurons, which agrees with Ansari et al. (74) and Adarmanabadi et al. (109). Furthermore, glutamate is a major neurotransmitter, and brain neurons have a unique glutamine metabolism (110). The amount of glutamate in the brain of the infected mice was significantly increased at 9 p.i. This is consistent with Parekh et al. (111) and Miranda et al. (112) reported an increase of glutamine levels in the metabolite pool from brain extracts of *P. berghei*-infected mice associated with neurological and behavioral changes. These studies suggest that an imbalance in glutamate/glutamine metabolism may be relevant to cerebral malaria pathogenesis. Upon treatment, JRLE ameliorates the level of glutamine-glutamate relationship by enhancing the activation of glutamine transporter function in astrocytes. This finding highlights the neuroprotective properties of JRLE as noted by Sharma et al. (76), Liu et al. (105), and Yeh et al. (106).

## Conclusion

This study demonstrated that *P. berghei* infection leads to chronic infection and brain damage in the C57BL/6 strain of mice. Treatment with *Juglans regia* leaf extracts (JRLE) provided significant protection against brain injury and influenced the disease outcome positively. However, the study primarily focused on the brain, and the impact of JRLE on other organs remains to be explored. Future research should investigate the broader organ-specific effects of JRLE, as well as the molecular mechanisms underlying the parasite–host interaction. Additionally, studies evaluating the long-term safety and efficacy of JRLE in different *Plasmodium* models would be crucial for assessing its therapeutic potential in malaria treatment.

## Data Availability

The raw data supporting the conclusions of this article will be made available by the authors, without undue reservation.
